# Association of Aminoacyl-tRNA Synthetases Gene Polymorphisms with the Risk of Congenital Heart Disease in the Chinese Han Population

**DOI:** 10.1371/journal.pone.0110072

**Published:** 2014-10-13

**Authors:** Min Da, Yu Feng, Jing Xu, Yuanli Hu, Yuan Lin, Bixian Ni, Bo Qian, Zhibin Hu, Xuming Mo

**Affiliations:** 1 Department of Cardiothoracic Surgery, The Affiliated Nanjing Children’s Hospital of Nanjing Medical University, Nanjing, Jiangsu, P.R. China; 2 Department of Thoracic and Cardiovascular Surgery, The First Affiliated Hospital of Nanjing Medical University, Nanjing, Jiangsu, P.R. China; 3 Department of Epidemiology and Biostatistics, State Key Laboratory of Reproductive Medicine, Nanjing Medical University, Nanjing, Jiangsu, P.R. China; Kunming Institute of Zoology, Chinese Academy of Sciences, China

## Abstract

Aminoacyl-tRNA synthetases (ARSs) are in charge of cellular protein synthesis and have additional domains that function in a versatile manner beyond translation. Eight core ARSs (*EPRS, MRS, QRS, RRS, IRS, LRS, KRS, DRS*) combined with three nonenzymatic components form a complex known as multisynthetase complex (MSC).We hypothesize that the single-nucleotide polymorphisms (SNPs) of the eight core ARS coding genes might influence the susceptibility of sporadic congenital heart disease (CHD). Thus, we conducted a case-control study of 984 CHD cases and 2953 non-CHD controls in the Chinese Han population to evaluate the associations of 16 potentially functional SNPs within the eight ARS coding genes with the risk of CHD. We observed significant associations with the risk of CHD for rs1061248 [G/A; odds ratio (OR) = 0.90, 95% confidence interval (CI) = 0.81–0.99; *P* = 3.81×10^−2^], rs2230301 [A/C; OR = 0.73, 95%CI = 0.60–0.90, *P* = 3.81×10^−2^], rs1061160 [G/A; OR = 1.18, 95%CI = 1.06–1.31; *P* = 3.53×10^−3^] and rs5030754 [G/A; OR = 1.39, 95%CI = 1.11–1.75; *P* = 4.47×10^−3^] of *EPRS* gene. After multiple comparisons, rs1061248 conferred no predisposition to CHD. Additionally, a combined analysis showed a significant dosage-response effect of CHD risk among individuals carrying the different number of risk alleles (*P*_trend_ = 5.00×10^−4^). Compared with individuals with “0–2” risk allele, those carrying “3”, “4” or “5 or more” risk alleles had a 0.97-, 1.25- or 1.38-fold increased risk of CHD, respectively. These findings indicate that genetic variants of the *EPRS* gene may influence the individual susceptibility to CHD in the Chinese Han population.

## Introduction

Congenital heart disease(CHD) is the most common human birth defect and the leading cause of perinatal mortality, with an incidence of approximately 6–8 per 1000 live births or even higher [1], [2], [3]. With the advances in surgical techniques, the prognosis of children with complicated and uncomplicated CHDs continues to improve, but the reported incidence remains unchanged [4]. The etiology of CHD is complex and possibly includes the interaction of inherited factors and environmental exposures [5], [6], [7]. A multitude of research studies have identified both chromosomal abnormality and gene mutations as causation for the syndromic heart malfunction [8]. However, the origin of non-syndromic CHD, which accounts for most of all congenital cardiac abnormalities, is waiting to be uncovered further.

Over the past decades, plenty of genes have been identified as candidates to be responsible for CHD [9], [10], [11]. However, aminoacyl-tRNA synthetases (ARSs) that seemed to be in charge of only cellular protein synthesis were overlooked. ARSs catalyze the attachment of amino acids to their cognate tRNAs with high fidelity [12], [13]. Recent research has shown that eukaryote ARSs, distinguished from their prokaryotic counterparts, have additional domains and motifs such as glutathione S-transferase (GST), WHEP domains, leucine zipper domains, and α-helicalappendices that function beyond translation [14] and may link with a variety of human diseases, such as cancer, neuronal pathologies, autoimmune disorders, and disrupted metabolic conditions [13], [15]. Recently, the nontranslational functions of vertebrate ARSs have been associated with cytoplasmic forms and nuclear and secreted extracellular forms that impact cardiovascular development pathways [16].

Eight core aminoacyl-tRNA synthetases (ARSs), bifunctional glutamyl-prolyl-tRNA synthetase (*EPRS*), isoleucyl-tRNA synthetase (*IRS*), leucyl-tRNA synthetase (*LRS*), methionyl-tRNA synthetase (*MRS*), glutaminyl-tRNA synthetase (*QRS*), lysyl-tRNA synthetase (*KRS*), aspartyl-tRNA synthetase (*DRS*), and arginyl-tRNA synthetase (*RRS*), form a macromolecular protein complex with three auxiliary factors, designated ARS-interacting multifunctional protein 1 (AIMP1), AIMP2 and AIMP3. This complex is known as the multisynthetase complex (MSC). The MSC may act as a depot for ARSs, which could be subsequently released from the macromolecular complexes to participate in auxiliary tasks beyond translation [17], generate a channel for the delivery of tRNAs [18], [19] and help the proofreading of newly synthesized nuclear tRNAs in the nucleus [20].

According to the expressed sequence tags (EST) profile in the public database UniGene (http://www.ncbi.nlm.nih.gov/UniGene), all eight of the core ARS coding genes were expressed in human heart tissues, with transcripts ranging from 44 to 502 per million (**Figure S1**). Thus, it is plausible that changes in the core ARSs may affect heart development and are related to the occurrence of CHD. However, to date, no research has reported a relation between the genetic variants of the core ARS genes and CHD susceptibility.

To determine the effect of genetic variants in the core ARS genes on CHD development, we conducted a case-control study by investigating the genotype frequency distribution of the 16 potential functional polymorphisms in the eight members of the MSC.

## Materials and Methods

### Ethics Statement

This study was approved by the institutional review board of Nanjing Medical University and adhered to the tenets of the Declaration of Helsinki. The design and performance of the current study involving human subjects were clearly described in a research protocol. All participants and/or their parents were voluntary and completed the informed consent in writing before taking part in this research.

### Study populations

The case-control analysis included 984 affected children with sporadic CHD and 2953 unrelated non-CHD controls. All subjects were genetically unrelated ethnic Han Chinese. Subjects for the study were consecutively recruited from the Affiliated Nanjing Children’s Hospital of Nanjing Medical University and the First Affiliated Hospital of Nanjing Medical University, Nanjing, China, from March 2009 to December 2011. All CHD patients were diagnosed based on echocardiography, with some diagnoses further confirmed by cardiac catheterization and/or surgery. Potential study subjects were initially surveyed with a brief questionnaire at clinics to determine whether they were willing to participate in a research study; we then conducted a face-to-face interview to obtain demographic information. Cases that had clinical features of developmental syndromes, multiple major developmental anomalies or known chromosomal abnormalities were excluded. The exclusion criteria also included a positive family history of CHD in a first-degree relative (parents, siblings and children), maternal diabetes mellitus, phenylketonuria, maternal teratogen exposure (e.g., pesticides and organic solvents), and maternal therapeutic drug exposure during the intrauterine period. Controls were non-CHD outpatients from the same geographic areas. They were recruited from the hospitals listed above during the same time period. Controls with congenital anomalies or cardiac disease were excluded. For each participant, approximately 2 ml of whole blood was obtained to extract genomic DNA for genotyping analysis.

### SNP selection and genotyping

Eight ARSs (*EPRS, MRS, QRS, RRS, IRS, LRS, KRS, DRS*) that formed MSC were selected. For each ARS-coding gene, we first used the public HapMap single nucleotide polymorphism (SNP) database (phase II+ III Feb 09, on NCBI B36 assembly and dbSNP b126) to search for SNPs that localized within gene regions, with MAF≥0.05, in the Chinese Han population. Then, a web-based analysis tool was used to predict the function of these SNPs (http://snpinfo.niehs.nih.gov/snpinfo/snpfunc.htm). Finally, a total of 27 potentially functional SNPs were selected in 8 ARS-coding genes. We next conducted linkage disequilibrium (LD) analysis by the Haploview 4.2 software, and only one SNP was selected in the case of multiple SNPs in the same haplotype block (*r^2^*>0.8). Eighteen (rs1061160, rs1061248, rs2230301 and rs5030754 in *EPRS*; rs508904 in *MRS*; rs193466, rs2305737 and rs244903 in *RRS*; rs1058751, rs10820966 and rs556155 in *IRS*; rs10988 in *LRS*; rs2233805 and rs3784929 in *KRS*; rs2164331, rs309142, rs309143 and rs6738266 in *DRS*) of 27 SNPs remained. Two SNPs (rs6738266 and rs2164331) were excluded due to primer design failure.

Genomic DNA was isolated from leukocyte pellets of venous blood by proteinase K digestion, followed by phenol-chloroform extraction and ethanol precipitation. Nanodrop and DNA electrophoresis were used to check the quality and quantity of DNA samples before genotyping. The genotyping was performed by Illumina Infinium BeadChip (Illumina, Inc.). All SNPs were successfully genotyped with call rates >95% (**Table 1**).

**Table 1:** Primary information for 16 functional SNPs in ARS-coding genes.

Gene	ARS	Chr. (cytoband)	SNP	Position (bp)^a^	Location	Predicted function^b^	MAF^c^	Allele^c^	HWE^d^	Genotyping call rate (%)
*EPRS*	Glutamyl-prolyl-tRNA synthetase	1q41	**rs1061248**	219968681	3′UTR	miRNA^e^	0.378	G/A	0.63	99.5
			**rs1061160**	219981426	exon	Splice sites^f^	0.366	G/A	0.76	99.8
			**rs5030754**	219983387	exon	Splice sites	0.012	G/A	0.82	99.9
			**rs2230301**	220024283	exon	Splice sites, nsSNP^g^	0.073	A/C	0.90	99.9
*DRS*	Aspartyl-tRNA synthetase	2q21.3	rs309143	135956608	intron	TFBS^h^	0.207	A/G	0.40	99.8
			rs309142	1395957754	intron	TFBS	0.488	A/G	0.29	99.8
*LRS*	Leucyl-tRNA synthetase	5q32	rs10988	146120433	exon	nsSNP	0.280	G/A	0.04	99.9
*RRS*	Arginyl-tRNA synthetase	5q34	rs244903	168486505	exon	Splice sites, nsSNP	0.146	A/G	0.35	99.7
			rs193466	1684865598	intron	TFBS	0.305	A/G	0.58	99.7
			rs2305737	168519256	3′UTR	Splice sites, miRNA	0.146	C/A	0.71	99.8
*IRS*	Isoleucyl-tRNA synthetase	9q22.31	rs1058751	92210634	3′UTR	miRNA	0.122	A/T	0.22	99.5
			rs556155	92223355	exon	nsSNP	0.195	A/G	0.25	99.9
			rs10820966	92293147	intron	TFBS	0.078	A/T	0.16	99.9
*MRS*	Methionyl-tRNA synthetase	12q13.3	rs508904	57488311	intron	TFBS	0.289	A/G	0.70	99.8
*KRS*	Lysyl-tRNA synthetase	16q23.1	rs3784929	75643129	intron	TFBS	0.159	G/A	0.66	99.8
			rs2233805	75647244	intron	TFBS	0.049	A/G	1.00	99.9

a Derived from the UCSC Genome Browser on Human Feb. 2009 (GRCh37/hg19) Assembly (http://genome.ucsc.edu/);

b Derived from an online tool-SNPinfo (http://snpinfo.niehs.nih.gov/snpfunc.htm);

c Major/minor allele;

d Hardy-Weinberg equilibrium test among controls;

e miRNA: microRNA

f Splice sites: Exonic splicing enhancer (ESE) or exonic splicing silencer (ESS) binding sites;

g nsSNP: non-synonymous polymorphisms.

h TFBS: Transcription factor binding sites;

### Statistical analyses

The differences between the CHD patients and control subjects were evaluated in the distributions of demographic characteristics, selected variables, and frequencies of genotypes of the 16 polymorphisms using Student’s t-test (for continuous variables) or the χ^2^ test (for categorical variables). The χ^2^ test determined the Hardy-Weinberg equilibrium of the genotype distribution of polymorphisms in the control group. LD between SNPs was evaluated using Haploview 4.2.Odds ratios (ORs) and 95% confidence intervals (CIs) were estimated by logistic regression analyses in the additive model to estimate the associations between the variants genotypes and risk of CHD. Chi-square-based Q-test was applied to test the heterogeneity of associations between subgroups, and the heterogeneity was considered significant when *P*<0.05. All statistical analyses were performed using the Statistical Analysis System software (v.9.1.3; SAS Institute, Cary, NC, USA). All tests were two-sided, and *P*<0.05 was considered significant.

## Results

**Figure 1:**
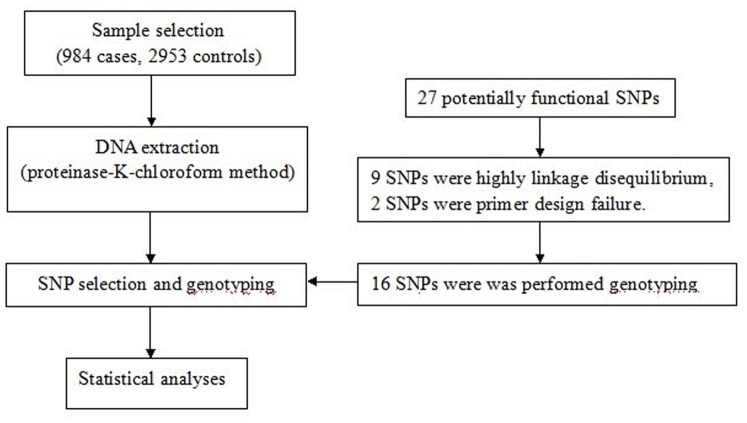
Study design procedures for association of ARSs gene polymorphisms with the risk of congenital heart disease in the Chinese Han population.

An overview of the study design using a flowchart was performed as shown in **Figure 1**. We systematically investigated the association of potentially functional SNPs with CHD susceptibility in 984 cases and 2953 controls in a Chinese population. There were no statistically significant differences for the distributions of age and gender between cases and controls (*P* = 0.261 and *P* = 0.832, respectively). Among the 984 CHD patients, 312 had atrial septal defect (ASD), 585 were diagnosed with ventricular septal defect (VSD), and 87 were diagnosed with ASD combined with VSD.

The genotype distributions of the 16 SNPs and the associations with CHD risk are summarized in **Table 2**. The observed genotype frequencies of these SNPs were in agreement with Hardy-Weinberg equilibrium in the controls (*P* value from 0.16 to 1.00) except rs10988 (*P* = 0.04). Among the 16 SNPs, significant associations were observed between 4 SNPs (rs1061248, rs1061160, rs5030754 and rs2230301) and CHD risk following a logistic regression analysis in the additive model. All four SNPs were in the *ERPS* gene. The G allele of rs1061248 and the A allele of rs2230301 were associated with a decreased risk of CHD [additive model: odds ratio (OR) = 0.90, 95% confidence interval (CI) = 0.81–0.99, *P* = 3.81×10^−2^; and OR = 0.73, 95%CI = 0.60–0.90, *P* = 3.53×10^−3^, respectively]; however, the G allele of rs1061160 and the G allele of rs5030754 were associated with an increased risk of CHD (OR = 1.18, 95%CI = 1.06–1.31, *P* = 1.28×10^−3^; and OR = 1.39, 95%CI = 1.11–1.75, *P* = 4.47×10^−3^, respectively). We further calculated *P* values for the false discovery rate to perform multiple comparisons. After comparisons, we found that rs2230301, rs5030754 and rs1061160 correlated with CHD risk, whereas rs1061248 lost its significant association with the risk of CHD. In contrast, no obvious evidence of a significant association between the other 12 SNPs and CHD risk was found.

**Table 2:** Summary of associations between 16 SNPs of MSC genes with congenital heart disease.

Chr.	Gene	SNP	Allele^a^	Case^b^ (N = 984)	Control^b^ (N = 2953)	MAF^c^		HWE^d^	Additive model		*P*_FDR_ e
(cytoband)						Cases	Controls		OR(95%CI)	*P*	
1q41	*EPRS*	**rs1061248**	G/A	217/486/271	741/1459/744	0.47	0.50	0.63	**0.90 (0.81–0.99)**	**3.81×10** ^−**2**^	**0.152**
		**rs1061160**	G/A	184/486/311	465/1402/1083	0.44	0.40	0.76	**1.18 (1.06–1.31)**	**1.82×10** ^−**3**^	**0.029**
		**rs5030754**	G/A	1/113/869	6/241/2704	0.06	0.04	0.82	**1.39 (1.11–1.75)**	**4.47×10** ^−**3**^	**0.023**
		**rs2230301**	A/C	3/114/865	20/440/2490	0.06	0.08	0.90	**0.73 (0.60–0.90)**	**3.53×10** ^−**3**^	**0.028**
2q21.3	*DRS*	rs309143	A/G	38/308/634	100/927/1921	0.20	0.19	0.40	1.03 (0.91–1.17)	6.40×10^−1^	1.024
		rs309142	A/G	172/499/310	526/1472/949	0.43	0.43	0.29	1.01 (0.91–1.12)	9.11×10^−1^	0.911
5q32	*LRS*	rs10988	G/A	47/340/596	120/1041/1789	0.22	0.22	0.04	1.02 (0.90–1.16)	7.32×10^−1^	0.901
5q34	*RRS*	rs244903	A/G	15/213/754	50/622/2270	0.12	0.12	0.35	1.01 (0.87–1.18)	9.06×10^−1^	0.966
		rs193466	A/G	74/399/509	229/1162/1553	0.28	0.28	0.58	1.02 (0.91–1.14)	7.72×10^−1^	0.505
		rs2305737	C/A	20/270/691	60/744/2145	0.16	0.15	0.71	1.10 (0.95–1.26)	2.12×10^−1^	0.565
9q22.31	*IRS*	rs1058751	A/T	0/150/828	11/412/2516	0.08	0.07	0.22	1.04 (0.86–1.27)	6.71×10^−1^	0.976
		rs556155	A/G	16/206/760	34/627/2291	0.12	0.12	0.25	1.03 (0.88–1.21)	6.79×10^−1^	0.905
		rs10820966	A/T	10/213/760	40/681/2230	0.12	0.13	0.16	0.91 (0.77–1.06)	2.21×10^−1^	0.505
12q13.3	*MRS*	rs508904	A/G	105/442/433	301/1268/1380	0.33	0.32	0.70	1.07 (0.96–1.20)	2.01×10^−1^	0.643
16q23.1	*KRS*	rs3784929	G/A	33/302/644	95/849/2005	0.19	0.18	0.66	1.08 (0.95–1.23)	2.40×10^−1^	0.480
		rs2233805	A/G	0/31/951	1/106/2844	0.02	0.02	1.00	0.86 (0.57–1.29)	4.63×10^−1^	0.823

a Major/minor allele;

b Variant homozygote/Heterozygote/Wild type homozygote;

c Minor allele frequency among cases/controls;

d Hardy-Weinberg equilibrium test among controls.

e Multiple comparisons *P* values for false discovery rate.

We have listed the results of the genotypic association analysis in **Table 3**. In dominant genetic model, for rs1061160 and rs5030754 polymorphisms, AG+AA and AG+GG genotypes were associated with an increased risk of CHD compared with the GG genotype, respectively(OR = 1.25, 95%CI = 1.07–1.46; OR = 1.44, 95%CI = 1.14–1.82). For rs2230301 polymorphism, AC+CC genotypes were associated with a decreased risk of CHD compared with the AA genotype(OR = 0.73, 95% CI = 0.59–0.91).

**Table 3:** Summary of genotypic association analysis of four SNPs of the *EPRS gene* with congenital heart disease.

SNP	Genotype	Cases	Controls	OR (95%CI)	*P*
rs1061248	GG	271	744	1.00	–
	AG	486	1459	0.91 (0.77–1.09)	3.11×10^−1^
	AA	217	741	**0.80 (0.65–0.99)**	**3.74×10** ^−**2**^
	AG+AA	703	2200	0.88 (0.75–1.03)	1.15×10^−1^
rs1061160	GG	311	1083	1.00	**–**
	AG	486	1402	**1.21 (1.03–1.42)**	**2.35×10** ^−**2**^
	AA	164	465	**1.38 (1.11–1.70)**	**3.07×10** ^−**3**^
	AG+AA	650	1867	**1.25 (1.07–1.46)**	**4.54×10** ^−**3**^
rs5030754	GG	869	2704	1.00	**–**
	AG	113	241	**1.46 (1.15–1.85)**	**1.72×10** ^−**3**^
	AA	1	6	0.52 (0.06–4.31)	5.44×10^−1^
	AG+GG	114	247	**1.44 (1.14–1.82)**	**2.51×10** ^−**3**^
rs2230301	AA	865	2490	1.00	**–**
	AC	114	440	**0.75 (0.60–0.93)**	**8.99×10** ^−**3**^
	CC	3	20	0.43 (0.13–1.46)	1.76×10^−1^
	AC+CC	117	460	**0.73 (0.59–0.91)**	**4.90×10** ^−**3**^

Additionally, we performed haplotype analysis (**Table 4**). As shown, the haplotype “GAAA” (combination of risk alleles of the four SNPs) was associated with an increased risk of CHD, whereas the protective allele combination “AGGC” was associated with a decreased risk of CHD. In the stratification analysis, we further evaluated the associations of the four SNPs in *EPRS* with CHD risk in subgroups stratified by gender and specific CHD phenotypes. As shown in **Table 5**, similar effects were observed among the subgroups.

**Table 4:** The haplotypic association of the four SNPs of the *EPRS* gene with congenital heart disease.

Haplotype^a^	case (%)	control (%)	OR (95%CI)	*P*
AGGA	905 (45.99)	2835 (48.0)	1.00 (referent)	
GAGA	740 (37.60)	2075 (35.13)	1.12 (0.99–1.25)	5.34×10^−2^
GGGC	100 (5.08)	370 (6.26)	0.85 (0.67–1.07)	1.62×10^−1^
GGGA	84 (4.27)	259 (4.39)	1.02 (0.79–1.31)	9.04×10^−1^
GAAA	113 (5.74)	253 (4.28)	**1.40 (1.11–1.77)**	**4.91×10** ^−**3**^
AGGC	20 (1.02)	107 (1.81)	**0.59 (0.36–0.95)**	**3.00×10** ^−**2**^
Others	6 (0.30)	7 (0.12)	2.69 (0.90–8.01)	7.65×10^−2^

a SNP order: rs1061248, rs1061160, rs5030754 and rs2230301.

**Table 5:** Stratified analysis on the associations between four SNPs in *EPRS* with congenital heart disease.

Characteristics	rs1061248					rs1061160				
	Case a	Control a	OR (95%CI) b	*P* b	*P* c	Case a	Control a	OR (95%CI) b	*P* b	*P* c
Gender										
male	105/241/138	472/856/464	0.87 (0.76–1.00)	4.90×10^−2^	0.527	91/237/160	286/844/667	1.16 (1.00–1.33)	4.79×10^−2^	0.751
female	112/245/133	269/603/280	0.93 (0.80–1.09)	3.74×10^−1^		93/249/151	179/558/416	1.20 (1.03–1.40)	1.83×10^−2^	
Diagnostic groups										
ASD	71/139/98	741/1459/744	0.84 (0.71–0.99)	4.27×10^−2^	0.483	68/146/98	465/1402/1083	1.26 (1.07–1.49)	6.44×10^−3^	0.489
VSD	126/299/154	741/1459/744	0.91 (0.80–1.03)	1.41×10^−1^		104/294/184	465/1402/1083	1.16 (1.02–1.32)	2.21×10^−2^	
ASD/VSD	20/48/19	741/1459/744	1.03 (0.76–1.39)	8.71×10^−1^		12/46/29	465/1402/1083	1.03 (0.76–1.40)	8.52×10^−1^	
**Characteristics**	**rs5030754**					**rs2230301**				
	**Case** a	**Control** a	**OR(95%CI)** b	***P*** b	***P*** c	**Case** a	**Control** a	**OR(95%CI)** b	***P*** b	***P*** c
Gender										
male	1/58/429	4/135/1659	1.58 (1.16–2.15)	3.90×10^−3^	0.211	3/64/421	13/280/1504	0.83 (0.63–1.09)	1.77×10^−1^	0.260
female	0/55/440	2/106/1045	1.18 (0.84–1.65)	3.37×10^−1^		0/50/444	7/160/986	0.65 (0.47–0.90)	9.74×10^−3^	
Diagnostic groups										
ASD	0/42/270	6/241/2704	1.62 (1.15–2.27)	5.54×10^−3^	0.119	0/31/280	20/440/2490	0.59 (0.41–0.86)	5.93×10^−3^	0.281
VSD	1/67/516	6/241/2704	1.40 (1.07–1.85)	1.56×10^−2^		2/76/506	20/440/2490	0.83 (0.65–1.06)	1.37×10^−1^	
ASD/VSD	0/4/83	6/241/2704	0.53 (0.19–1.43)	2.08×10^−1^		1/7/79	20/440/2490	0.62 (0.31–1.21)	1.62×10^−1^	

a Major/minor allele;

b Calculated by additive model;

c *P* for heterogeneity.

We also conducted a combined analysis of the four promising SNPs to test their joint effects on CHD risk. There was a significant dosage-response effect among individuals carrying the different number of risk alleles and CHD risk (*P*_trend_ = 5.00×10^−4^). Compared with individuals with “0–2” risk allele, those carrying “3”, “4” or “5 or more” risk alleles had a 0.97- (95% CI = 0.69–1.37), 1.25- (95% CI = 1.05–1.50) or 1.38-fold (95% CI = 1.14–1.68) increased risk of CHD, respectively (**Table 6**).

**Table 6:** Combined effects of rs1061248, rs1061160, rs1061160, and rs2230301 on CHD.

Number of risk alleles^a^	Case (%)	Control (%)	OR (95% CI)^b^	*P* b
0–2	262 (26.95)	939 (31.96)	1.00	
3	50 (5.14)	184 (6.26)	0.97 (0.69–1.37)	8.79×10^−1^
4	384 (39.51)	1098 (37.37)	1.25 (1.05–1.50)	1.37×10^−2^
≥5	276 (28.40)	717 (24.40)	1.38 (1.14–1.68)	1.20×10^−3^
Trend				5.00×10^−4^

a rs1061248 G, rs1061160 A, rs5030754 A, and rs2230301 A were assumed as risk alleles;

b Calculated by additive model.

## Discussion

In this study, we systematically investigated the association of potentially functional SNPs in ARS-coding genes of the MSC with CHD susceptibility in 984 cases and 2953 controls in a Chinese population. We observed significant association of four SNPs (rs1061248, rs1061160, rs5030754 and rs2230301) in the *EPRS* gene with the risk of CHD, and the risk remarkably accelerated in the individuals who carried more risk alleles. Although ASD and VSD represent the most common congenital heart malfunctions, the accurate pathogenesis is poorly understood. Based on previous research, the ARS-coding genes of MSC take part in diverse functional activities, and some of them have been proven to be crucial for heart development and proper functioning. Few studies have linked the variants of MSC genes to congenital heart disease. To our knowledge, we provide the first evidence that SNPs in *EPRS*, one of the core coding genes in MSC, may modulate the process of CHD.

Some ARSs in MSC have been demonstrated to have a close correlation with cardiovascular development. Glutamyl-prolyl-tRNA synthetase (*EPRS*) is a bifunctional enzyme that could translationally suppress vascular endothelial growth factor-A (VEGF-A) to regulate angiogenesis [21] and seems to act as a key gatekeeper of inflammatory gene translation [22]. Lysyl-tRNA synthetase (*KRS*) is secreted to trigger pro-inflammatory response [23] and plays a key role via Ap4A as an important signaling molecule in the transcriptional activity of microphthalmia transcription factor(MITF) [24], which has been demonstrated to be necessary in heart growth [25]. Glutaminyl-tRNA synthetase (*QRS*) can bind and inhibit the apoptotic activity of apoptosis signal-regulating kinase 1 (ASK1) [26], which has been demonstrated to be a new intracellular regulator of p38 MAPK activation in cardiac myogenic differentiation [27]. Han and colleagues [28] reported that Leucyl-tRNA synthetase (*LRS*) acts as a vital mediator for amino acid signaling to mTORC1, and the latter has been found to be related to the normal development of cardiovascular tissue [29].

Human *EPRS*, the largest polypeptide from the complex, is a bifunctional enzyme in which the two domains exhibiting each catalytic activity are linked by three tandem WHEP motifs [30]. *EPRS* contains 29 exons and 28 introns. In response to interferon-γ (IFNγ), *EPRS* is phosphorylated and released from its residence in the MSC. MSC then forms another multi-component complex, known as IFN-γ–activated inhibitor of translation (GAIT), with other regulatory proteins at a 3′UTR region that is involved in the translational silencing of target transcripts, such as VEGF-A [31], [32], [33]. As documented in many studies, VEGF-A shares a close relationship with CHD, and both the increased and decreased expression of VEGF-A during heart development can result in various CHD [34], [35], [36]. The SNP rs2230301, a missense SNP located at the 23rd exon of the *EPRS* gene, may act as a part of the exonic splicing enhancer based on the online tool SNPinfo [37]. The missense mutation would change the sequence of *EPRS* and may lead to protein misfolding and malfunction. We used a web-based analysis tool to predict the potential function of the SNPs, and rs2230301 was predicted to be a missense variant that may result in an amino acid alteration from aspartic acid (Asp) to glutamic acid (Glu) (http://snpinfo.niehs.nih.gov/snpinfo/snpfunc.htm). The NCBI database confirmed the results (http://www.ncbi.nlm.nih.gov/). However, the predicted results differed from the *in-silico* analysis. To further validate the function of this variant, some functional studies should be performed in some follow-up studies. The SNP rs1061248 is located at the 3′ regulatory region of the *EPRS* gene with a predicted function as a MicroRNA-binding site. Considering its potentially functional role, it is likely that this polymorphism might alter miRNA binding, thereby modulating the biological function of *EPRS*. The two synonymous SNPs rs1061160 and rs5030754 were localized on the seventh exon and the eleventh exon, respectively. Recently, a synonymous SNP was reported to alter the function of the protein in certain circumstances [38].

Several limitations of the present study need to be addressed. First, we did not replicate the results in additional individuals; this may contribute to potential false positive errors. The present analysis was restricted to individuals of Chinese Han descent, and therefore, the findings may not hold true for individuals of other races and ethnicities. Additionally, the limited sample size may contribute to the failed validation in the stratified analysis concerning the association between the SNPs and CHD. We performed the statistic power analysis of the significant SNPs in the studied population. The powers of three SNPs (rs1061248, rs5030754, and rs2230301) are lower than 0.6 because the sample size of our study is relatively small (984 CHD cases and 2953 non-CHD controls) and the effects of our target common SNPs are weak. Further replication of the association signal in an independent cohort for the four SNPs would support the conclusions. Therefore, the results are required to be further replicated by well-designed studies in additional large-scale Chinese Han populations.

In conclusion, we conducted a case-control study to investigate the role of genetic variants in ARS-coding genes of MSC in the development of CHD in a Chinese population. We observed that four SNPs (rs1061248, rs1061160, rs5030754, and rs2230301) in the *EPRS* gene may confer susceptibility to sporadic CHD and that the risk significantly increased with the number of risk alleles. However, further studies with functional evaluations are warranted to elucidate the potentially biological mechanisms of these polymorphisms in the development of CHD.

## Supporting Information

Figure S1 **Expressed sequence tags (EST) profile of the 8 core ARSs coding genes.**
(DOC)
