# Supplementary figures and images for: Association of Aminoacyl-tRNA Synthetases Gene Polymorphisms with the Risk of Congenital Heart Disease in the Chinese Han Population
Source: PLoS One. 2014 Oct 13;9(10):e110072. doi: 10.1371/journal.pone.0110072 (PMC4195700; doi:10.1371/journal.pone.0110072)

Supplementary Figure S1.Expressed sequence tags (EST) profile of the 8 core ARSs coding genes.


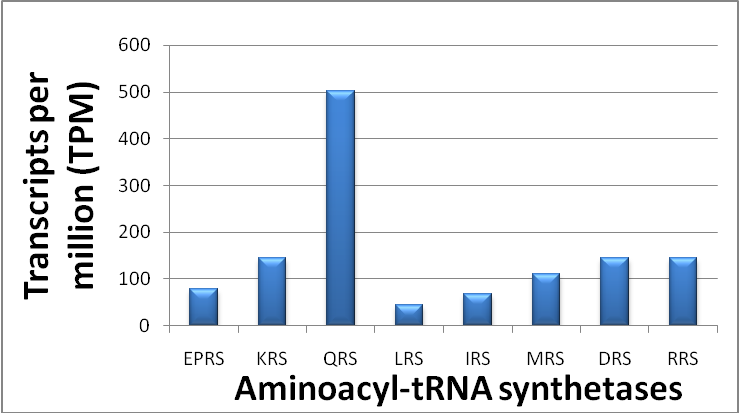

Supplement: Figure S1 — Expressed sequence tags (EST) profile of the 8 core ARSs coding genes. (DOC) [file pone.0110072.s001.doc]
